# Observations on seasonal occurrence in lymphomas with proven or presumed infectious etiology

**DOI:** 10.3389/fonc.2025.1586441

**Published:** 2025-07-08

**Authors:** Ioannis Anagnostopoulos, Viktoria Buck, Elena Gerhard-Hartmann, Alberto Zamó, Andreas Rosenwald, Mathias Rosenfeldt, Korinna Jöhrens

**Affiliations:** ^1^ Institute of Pathology, University of Würzburg, Würzburg, Germany; ^2^ Comprehensive Cancer Center Mainfranken, Würzburg, Germany; ^3^ Institute of Pathology, Klinikum Chemnitz gGmbH, Chemnitz, Germany

**Keywords:** seasonality, infectious etiology, classic Hodgkin lymphoma (cHL), marginal zone lymphomas, hairy cell leukemia, lymphocyte predominant Hodgkin lymphoma

## Abstract

**Introduction:**

There are relatively few studies on seasonal occurrence of lymphomas, most dealing with Hodgkin lymphoma (HL). Most studies were based on small datasets leading to partly conflicting patterns and results with only two using the large Surveillance, Epidemiology and End Results database. By doing this, the study on HL showed a peak incidence in March and the lowest in September, while the study on a broadly defined B-cell lymphoma group identified a peak in March and April. Among the hypotheses regarding seasonal occurrence of lymphomas, a potential infectious etiology has been proposed, at least for some patient subgroups.

**Methods:**

For the present study, we used the files from one of Germany’s lymphoma reference centers and addressed not only seasonal occurrence but also whether lymphoma entities with proven or suspected infectious etiology were associated with a particular seasonal clustering. We also investigated whether the COVID-19 pandemic (period 2020-21) influenced any observed seasonal patterns. Our study population comprised 8,038 cases with primary diagnosis of classic HL (CHL) including 2,434 cases with Epstein-Barr virus (EBV) infection of the neoplastic cells, 1,402 cases of nodular lymphocyte predominant HL, 487 cases with cutaneous marginal zone lymphoma (MZL), 247 cases with pulmonary MZL, 451 cases with hairy cell leukemia (HCL) and 4,577 cases with diffuse large B cell lymphoma (DLBCL).

**Results and discussion:**

Our data show that among HL only CHL exhibited seasonal fluctuation with a peak in the first quarter and a trough in the third quarter of the year. Similar seasonal patterns were observed in the nodular sclerosing CHL subtype and the younger patient age group (0–39 years). No seasonal fluctuation was identified in lymphomas with proven (EBV-positive CHL) or presumed infectious etiology (cutaneous and pulmonary MZL, HCL). COVID-19 pandemic did not significantly influence the seasonal occurrence patterns observed in CHL.

## Introduction

There are relatively few studies addressing the possibility of seasonal clustering of malignant lymphomas. Most have investigated Hodgkin lymphoma (HL) mainly in northern European countries. The majority of these studies have used small datasets and reported partly conflicting patterns and results (1–6). A more recent study of a large number of cases using the Surveillance, Epidemiology and End Results (SEER) database (www.seer.cancer.gov) not only identified a seasonal variation with a peak incidence in March and the lowest incidence in September, but also correlated this with overall survival (7). Regarding Non-Hodgkin lymphomas (NHL) the available data is sparse. The majority of studies, limited by small sample sizes and univariate statistical testing, have yielded null results (2, 6, 8), with only one study using the SEER database showing a peak in March and April in a broadly defined “B-cell-NHL” category in patients below age of 65 years (9).

Several hypotheses exist regarding the cause of the reported seasonal occurrence of lymphomas. Several authors have suggested that a potential infectious etiology may be a critical seasonal-related factor, at least in some subgroups of the patients (1, 5, 8–11). In the present study, we were interested in whether lymphomas with proven or suspected association with infectious etiology might show a similar seasonal variation. We were particularly interested in studying classic Hodgkin lymphomas (CHL) with and without Epstein-Barr virus (EBV) infection of the neoplastic cells (12). The association between EBV infection and CHL is not homogeneous. It varies between different age groups, geographical regions and histologic subtypes of this lymphoma (12). The EBV-infected neoplastic Hodgkin- and Reed-Sternberg cells exhibit a latency type II program, which among others includes expression of the EBV-encoded latent membrane proteins (LMP) 1 and 2A. LMP1 is an oncogene and mimics an active CD40 receptor, whereas LMP2A mimics B-cell receptor signaling, implying that these two viral proteins act as survival signals for the neoplastic cells (13, 14).

Another lymphoma group we included in this study is extranodal marginal zone lymphoma (MZL). These lymphomas occur most often in organs usually devoid of lymphocytes and their development has been linked to chronic infections and autoimmune conditions (15). We specifically analyzed MZLs with primary lung and skin manifestations, as both are organs with a range of frequent infectious and inflammatory processes. In addition, there is some but no definitive evidence for an association of these lymphomas with a particular microorganism. Regarding pulmonary MZL, one study could detect the presence of *Achromobacter xylosoxidans* in such cases (16), although this could not be confirmed in other studies (17, 18). Infection with *Borrelia burgdorferi*, the causative agent of Lyme disease, which is transmitted by tick bites (19) is known to cause a variety of skin disorders, and there is some evidence that genetic material of this spirochete can be associated with cutaneous MZL (20). We also included hairy cell leukemia (HCL), a rare indolent B-cell neoplasm involving the peripheral blood and diffusely infiltrating the bone marrow and splenic red pulp. Although it is characterized by a disease-defining genetic event, the *BRAF V600E* mutation in virtually all cases, some features suggest a possible association with chronic infection (21). One of these derives from the analysis of the immunoglobulin gene repertoire of this malignancy showing the presence of mutated immunoglobulin heavy chain variable region (*IGHV*) genes with evidence for antigen selection in the majority of cases. Further evidence comes from the immunophenotype of the HCL cell, including expression of CD11c (ITGAX, integrin subunit alpha X), T-bet (TBX21, T-box transcription factor 21) and PD1 (PDCD1, programmed cell death 1) but not of CD27 (22–24). This phenotype delineates a subset of B-cells present in the blood and splenic red pulp, but rarely in lymph nodes. Cells with a CD11c+, T-bet+ immunophenotype are found within B-cell populations that increase with age and are expanded in conditions associated with chronic antigenic stimulations such as infections with human immunodeficiency virus and malaria, and in autoimmune diseases such as systemic lupus erythematodes (25). In order to verify whether NHLs with suspected infectious etiology do indeed show a seasonal association at diagnosis we used as a control group one of the most frequent B-cell lymphoma entities, the diffuse large B-cell lymphoma (DLBCL), no other specified (NOS) that has no known association with an infection or chronic antigenic stimulation.

Another fact that interested us was whether the COVID-19 pandemic had any effect on the seasonal occurrence of the studied entities particularly trough the repeated and long lock-down periods.

## Methods

All data were retrospectively obtained from the files of the Reference center for Hematopathology at the Institute of Pathology, University of Würzburg, Germany. Since the reference center receives its specimens within short time after they have reached the local pathologists across Germany, we used the month of material receipt as the month of lymphoma/leukemia diagnosis. All diagnoses had been established according to the requirements of the current WHO classification for Hematolymphoid Tumors (26). In order to obtain representative sample sizes, we evaluated different time intervals for each disease entity. Specifically, we included all cases with a primary diagnosis of CHL established in the years 2007-2021, and for assessment of the much rarer nodular lymphocyte predominant Hodgkin lymphoma (NLPHL), cutaneous MZL, pulmonary MZL, and HCL we included all cases diagnosed within the time interval 2002-2021. Cases with recurrent lymphoma or residual disease after therapy were excluded. As the control group of DLBCL represents one of the most frequent lymphoma entities, we decided to include only cases diagnosed within a five-year interval (2015-2019).

Our study population included 8.038 cases with primary diagnosis of CHL encompassing 2.434 cases with an EBV infection of the neoplastic cells, 1.402 cases of NLPHL, 487 cases with cutaneous MZL, 247 cases with pulmonary MZL, 451 cases with HCL and 4577 cases with DLBCL. In addition, several subgroups were analyzed for each entity: sex (male, female), different age groups (0 – 19, 20 – 39, 40 – 59, 60 – 79, 80–100 years) as well as the possible influence of the COVID-19 pandemic during the period 2020-2021. For CHL, we also analyzed the information of different histologic subtypes (nodular sclerosis, mixed cellularity, lymphocyte-rich, lymphocyte-depleted) as well as the association with an EBV infection of the neoplastic cells.

For this study, we evaluated the number of primary diagnoses and the various investigated subgroups/features within a quarter of a year. Since the different quarters of the year exhibit different numbers of days, we divided the number of cases per quarter with the number of days per quarter and multiplied it with the mean number of days of all quarters of the year. Statistical analysis was performed using SPSS^®^. As a normal distribution of primary manifestation of malignancy could not be expected, we applied Mann-Whitney-U-Test to compare the numbers of primary manifestations between two quarters of a year. The assessment of significantly differing results in the various subgroups such as age, sex, histologic subtypes of CHL, EBV positive vs. EBV negative CHL etc. was performed using chi-square-test. A *P* value of less than 0.05 was considered statistically significant. Values between 0.05 and 0.10 were considered a trend. All statistical analyses were visualized using GraphPadPrism^®^.

## Results

### Classic Hodgkin lymphoma

**Figure 1:**
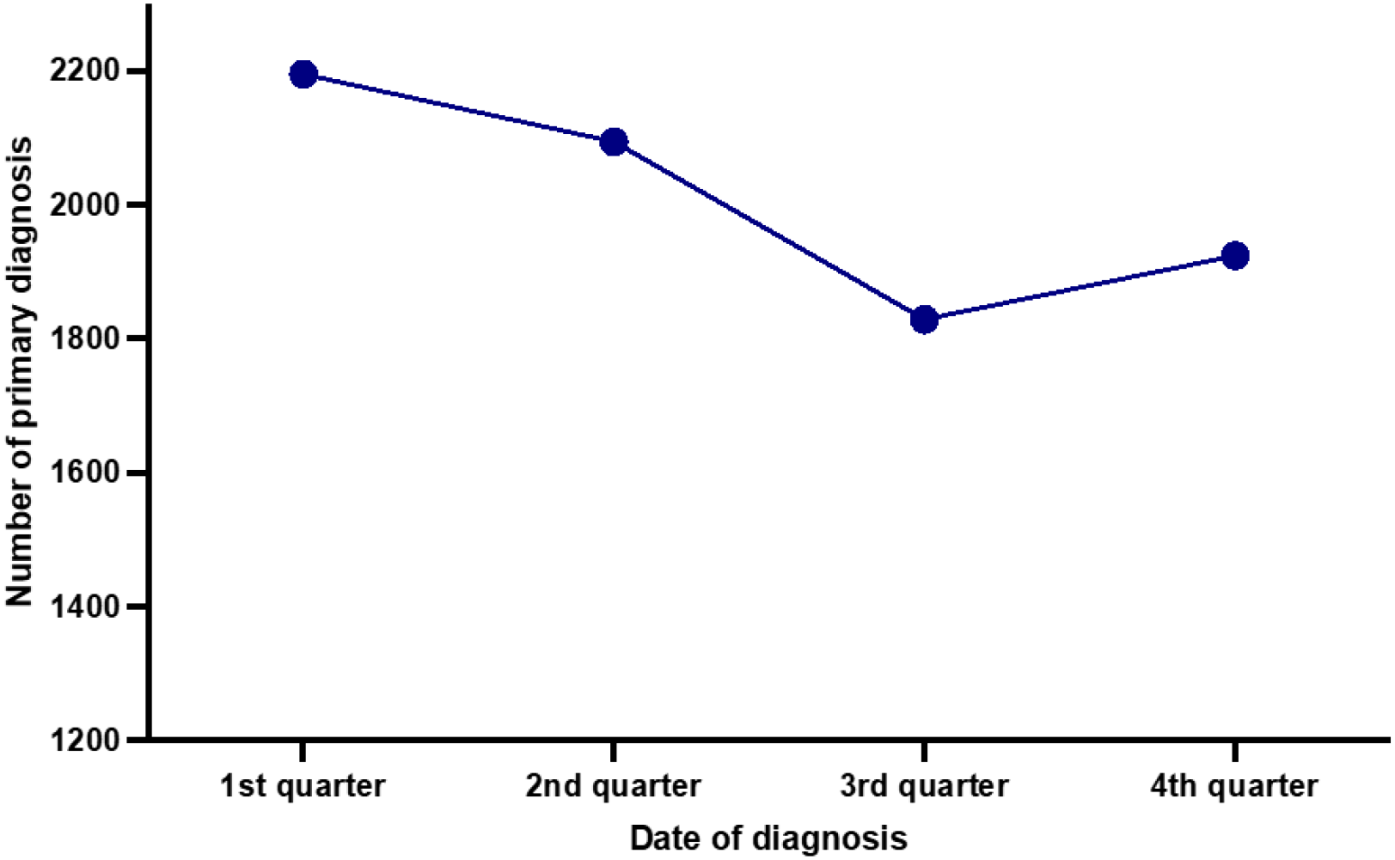
Seasonal distribution of newly diagnosed classic Hodgkin lymphoma cases in the years 2007-2021.

When all newly diagnosed cases were considered together, a cosinusoid pattern of primary diagnosis in the years 2007–2021 emerged, with a peak in the first quarter (27.3%) and a trough in the third quarter of the year (22.8%). There were significant differences in the numbers of newly diagnosed cases between the first and the third (p=0.003), the first and the fourth (p=0.033) as well as between the second and the third (p=0.005) quarter of the year. The highest difference was found between the first and the third quarter with a 20.0% higher incidence in the first quarter of the year (**Figure 1**).

**Figure 2:**
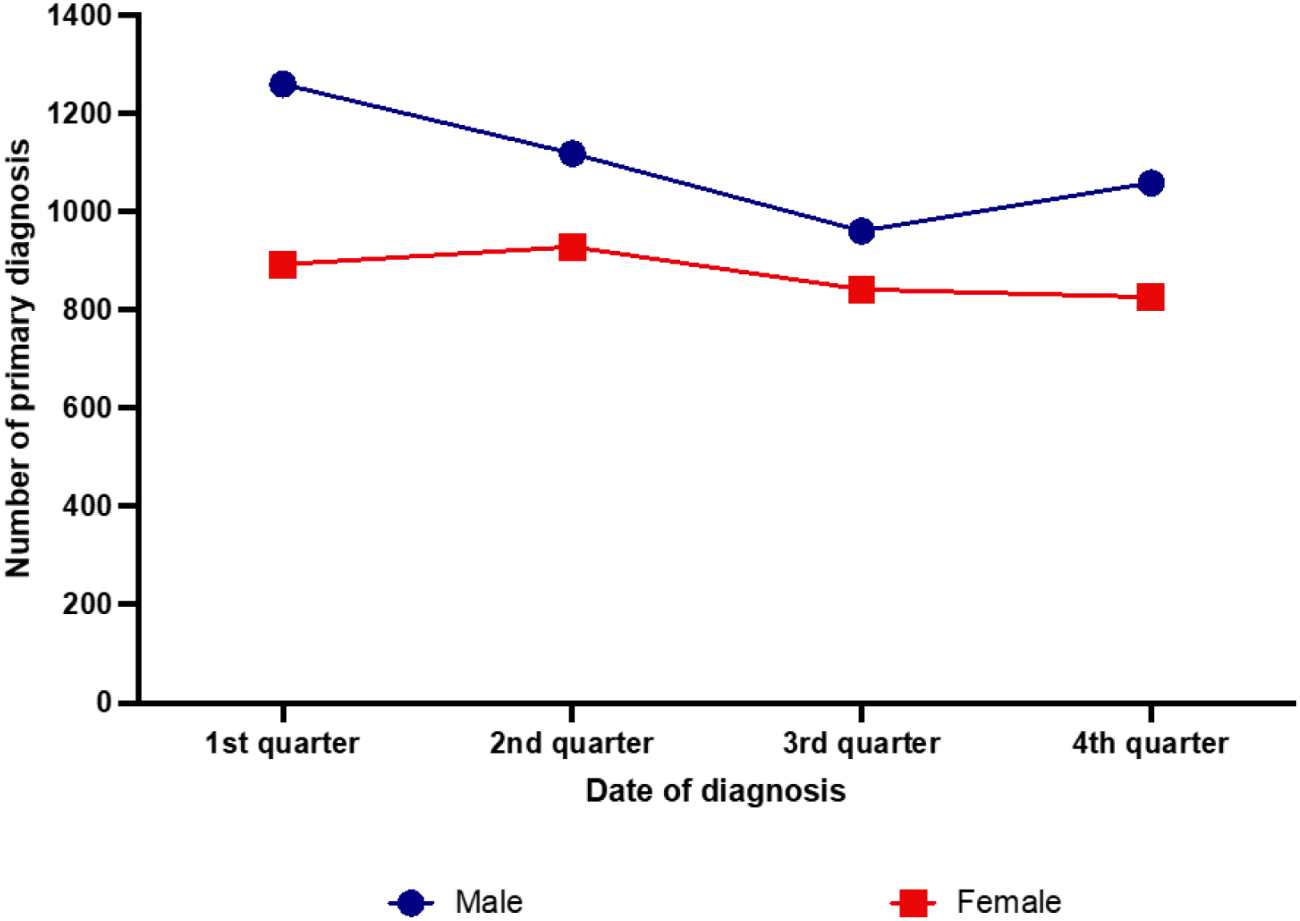
Seasonal distribution of newly diagnosed classic Hodgkin lymphoma cases in the years 2007–2021 according to the patients’ gender.

The observed cosinusoid seasonal pattern was present particularly in male patients (**Figure 2**). In the first quarter of the year, CHL was diagnosed in 28.6%, in the second in 25.4%, in the third in 21.8% and in the fourth in 24.1% of the male patients. The highest difference was observed between the first and third (p <0.001) quarter of the year. Significant differences were also found comparing the primary diagnoses in male patients between first and the second (p= 0.025), first and fourth (p <0.001), between second and third (p=0.012), and between third and fourth (p=0.048) quarters. The comparison of the seasonal patterns among male and female patients disclosed significant differences (p=0.007). The curve of the female cases was flatter and shifted to the right side. The proportion of primary diagnoses in the first quarter of the year was 25.6%, in the second 26.6%, in the third 24.1% and in the fourth 23.7%. A significant difference could be found only between second and fourth quarter (p=0.046). A potential confound regarding the proportion of lower numbers of diagnosis during the third quarter of the year might be caused by reduced diagnoses during summer holidays either due to patients’ delay and/or physicians’ absence

**Figure 3:**
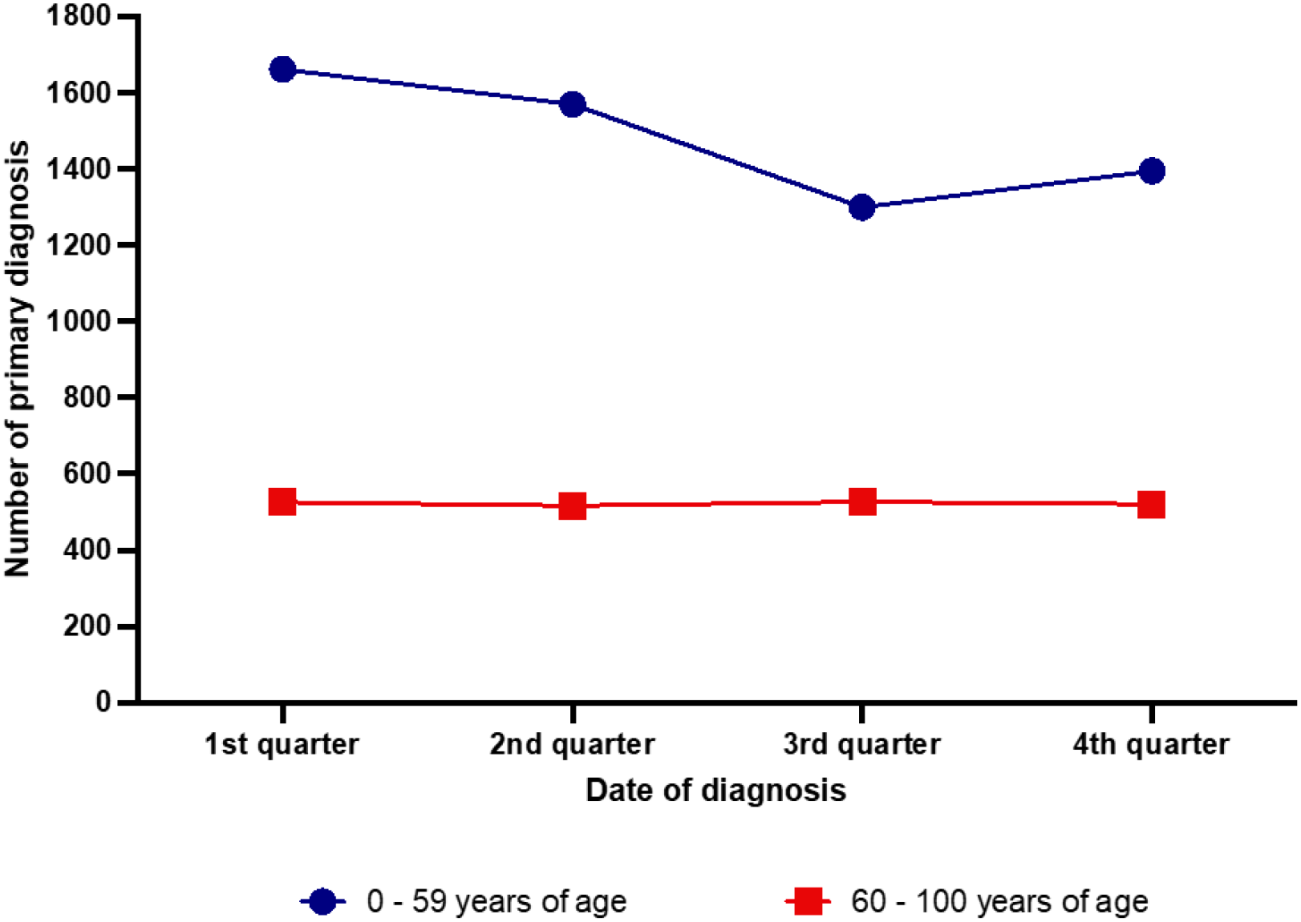
Seasonal distribution of newly diagnosed classic Hodgkin lymphoma cases in the years 2007–2021 according to the patients’ age.

Stratification by age groups (**Figure 3**) revealed a significant difference particularly between the younger (0–59 years of age) and the older (60–100 years of age) group (p=0.002). A seasonal pattern was present only in the younger age group showing 27.9% more newly diagnosed CHL in the first than in the third quarter of the year (p=0.001).

**Figure 4:**
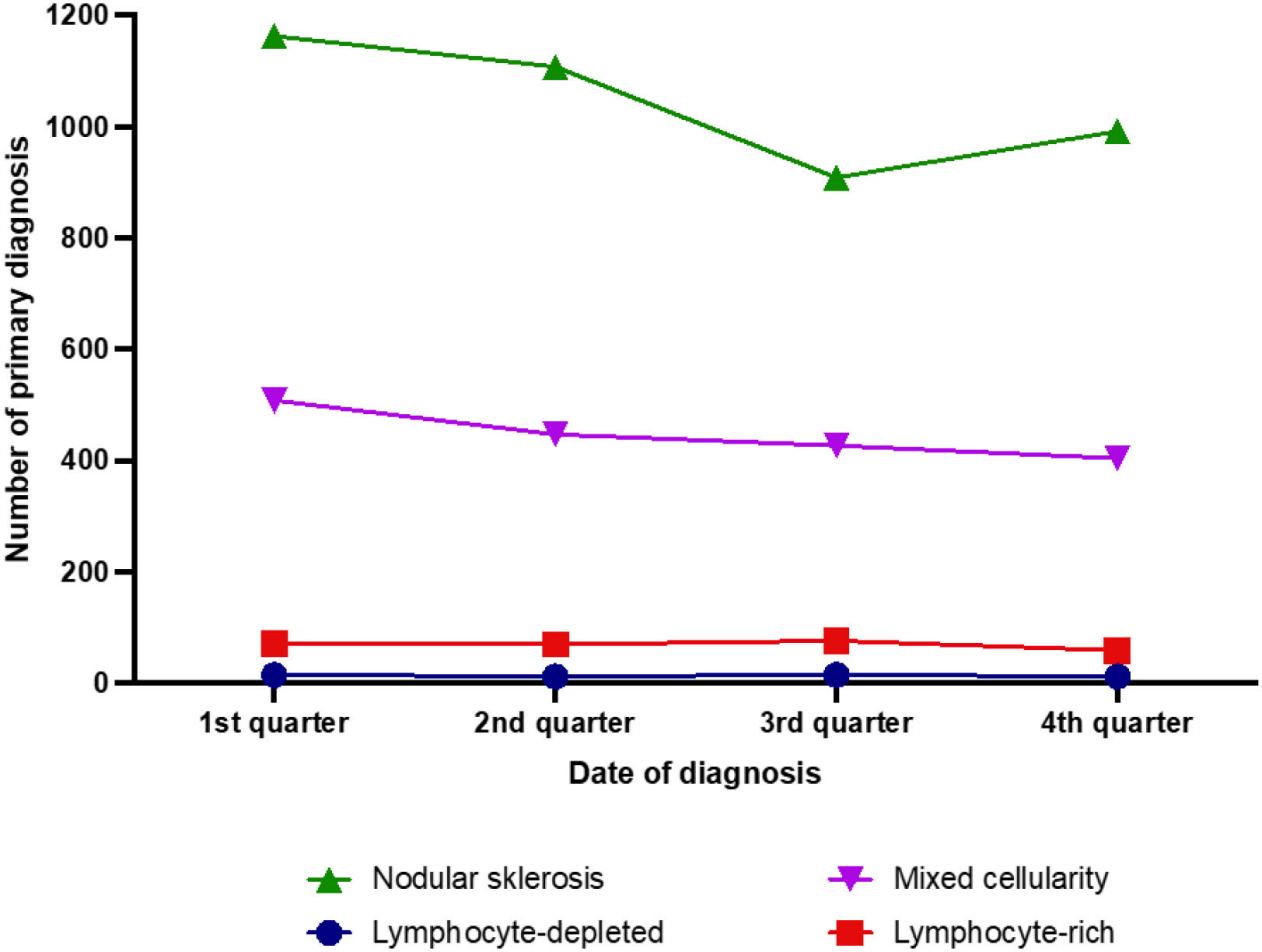
Seasonal distribution of newly diagnosed classic Hodgkin lymphoma cases in the years 2007–2021 according to histologic subtype.

Stratified by histological subtype only cases of nodular sclerosis showed a significant seasonal incidence that also exhibited the same cosinusoid pattern characterizing the overall seasonal incidence of CHL (**Figure 4**). This subtype exhibited significant differences between first and third (p=0.008), between second and third (p=0.017), and between first and fourth (p=0.038) quarter. Cases of mixed cellularity subtype displayed a significant difference between the first and the fourth quarter of the year (p = 0.0046), while the other subtypes did not show any significant seasonal differences; however, these subtypes included relatively small patient numbers (4169 cases of nodular sclerosis vs. 1784 mixed cellularity, 276 lymphocyte-rich and 54 lymphocyte depleted.

**Figure 5:**
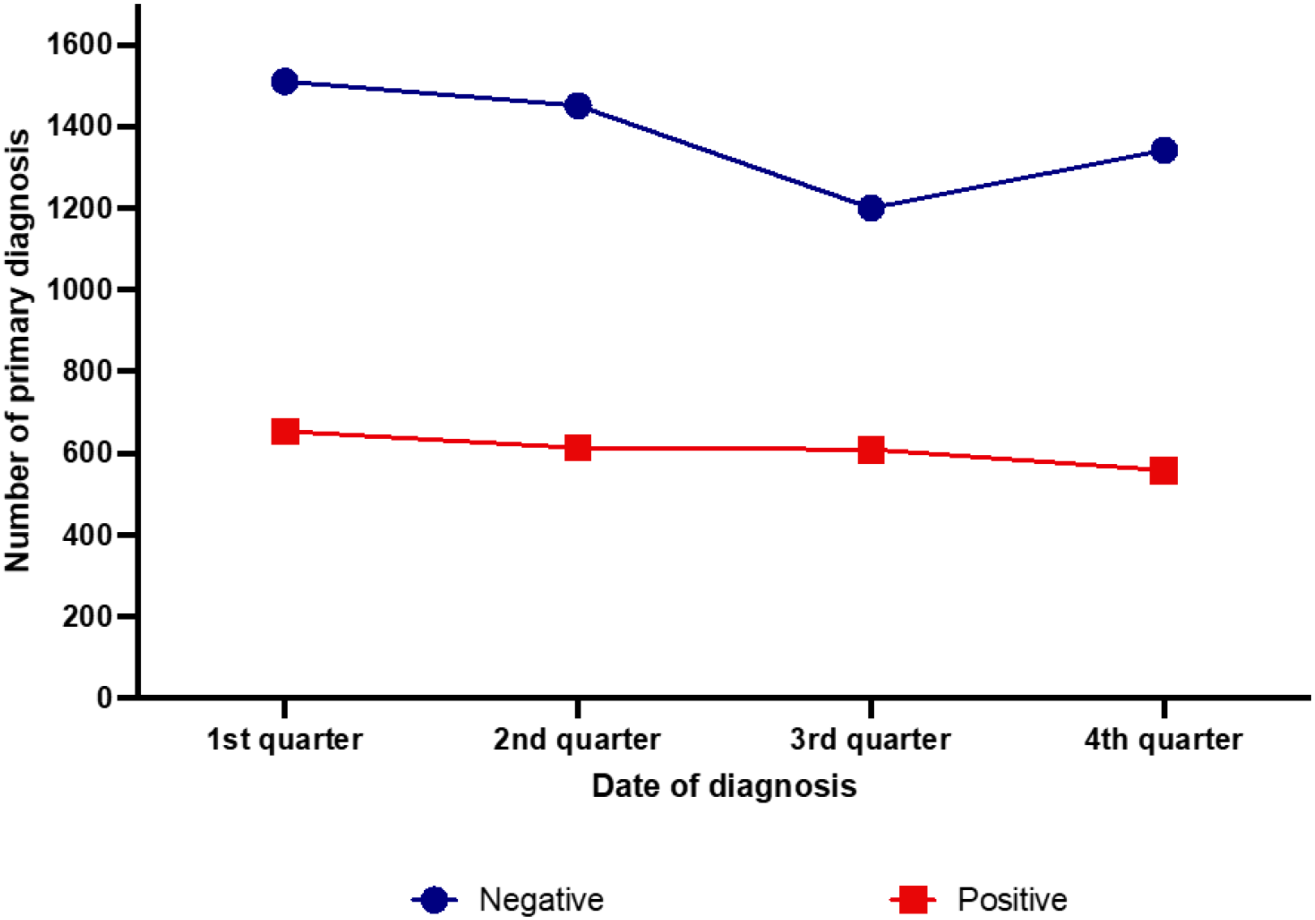
Seasonal distribution of newly diagnosed classic Hodgkin lymphoma cases in the years 2007–2021 according to the presence of Epstein-Barr virus-infected neoplastic cells.

Stratification of seasonal occurrence of primary diagnosis of EBV-positive and –negative CHL cases showed significant differences (p =0.016). The curve of the EBV-negative cases displayed a similar cosinusoid appearance as the total number of CHL cases with an incidence of 27.4% in the first quarter, 26.4% in the second, 21.8% in the third and 24.4% in the fourth quarter of the year (**Figure 5**). There were significant differences regarding the number of primary diagnoses between first and third (p <0.001), first and fourth (p =0.040) as well as between second and third (p < 0.001) and third and fourth quarter of the year (p=0.046). The EBV-associated cases showed a rather flat incidence curve with a sole significant difference between the first and the fourth quarter of the year (p=0.046).

**Figure 6:**
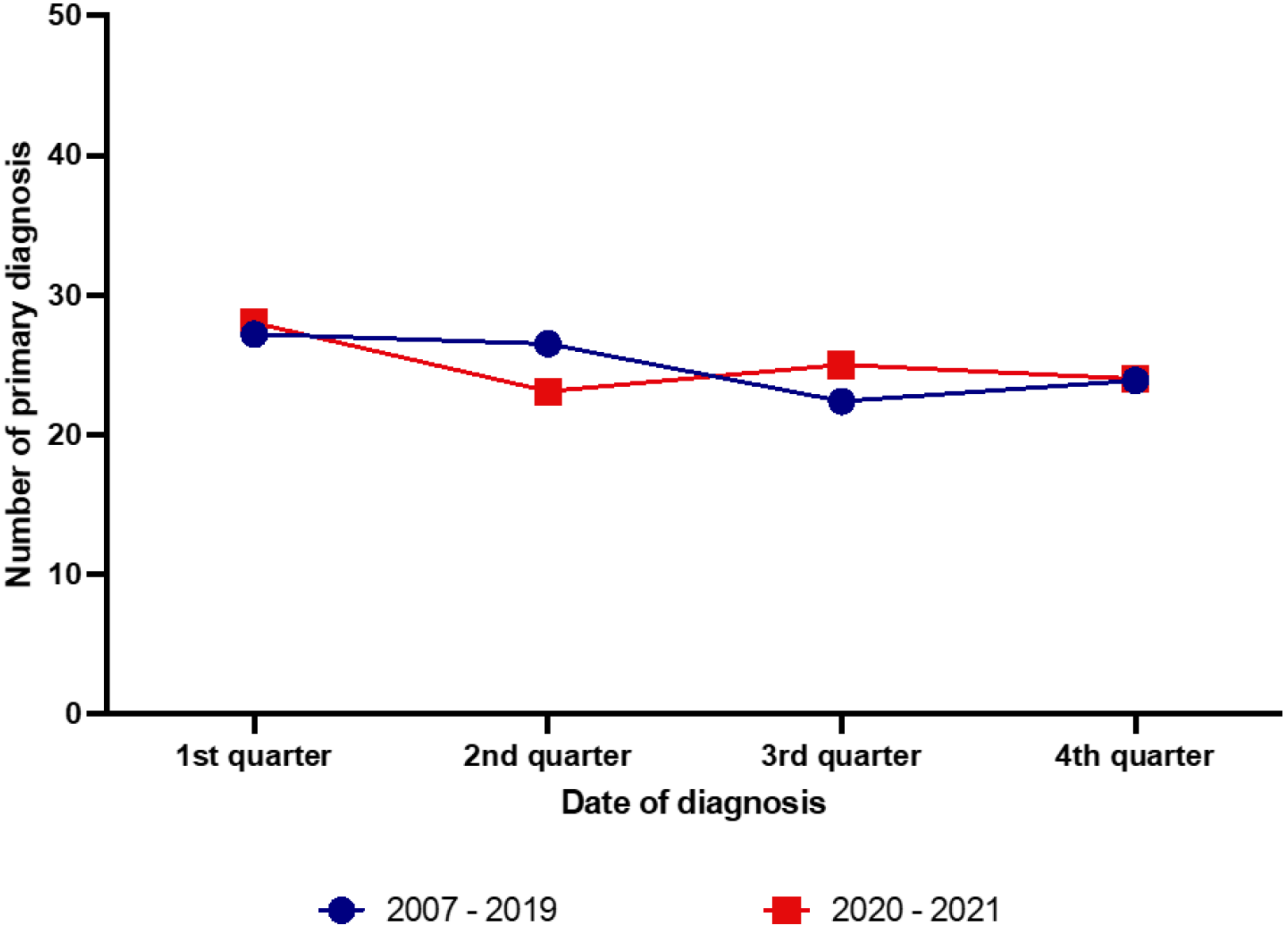
Comparison of the seasonal patterns of newly diagnosed classic Hodgkin lymphoma cases before (period 2007-2019) and during the COVID-19 pandemic (2020-2021).

Comparison of the seasonal patterns before (period: 2007-2019) and during the COVID-19 pandemic (2020-2021) showed some significant differences, in particular the observed cosinusoid incidence pattern disappeared during the pandemic period (**Figure 6**).

### Nodular lymphocyte predominant Hodgkin lymphoma

Primary diagnosis of NLPHL was established in 22.7% of the patients in the first, in 25.8% in the second, in 26.1% in the third and in 25.3% in the fourth quarter. These incidence variations were not statistically significant. Stratifications of the cases by age or sex did also not reveal any statistically significant seasonal differences. There were also not any differences between the periods before and during the COVID-19 pandemic.

### Cutaneous marginal zone lymphoma

The number of newly diagnosed cutMZL declines throughout the year without showing any statistically significant seasonal variations. 29.5% of the diagnoses were established in the first quarter, 24.1% in the second, 24.0% in the third and 22.4% in the fourth quarter of the year.

Stratification of the cases by sex showed significant differences between male and female patients (p=0.007). The incidence showed a continuous decline among male patients (33.5% in the first, 24.0% in the second, 26.0% in the third and 16.5% in the fourth quarter of the year respectively). The most significant difference occurred between the first and the fourth quarter of the year (p=0.020). The incidence curve of female patients did not show significant differences between the various quarters of the year (24.7% in the first, 24.5% in the second, 22.1% in the third and 28.7% in the fourth quarter).

Regarding the influence of age, no significant differences among the various groups could be verified. Only among the patients of the youngest age group (0–19 years) a significant (p=0.037) difference between the cases of the third and fourth quarters of the year was noted.

No statistically significant differences could be observed in the seasonal incidence patterns between the periods before and during the COVID-19 pandemic.

### Pulmonary marginal zone lymphoma

In the investigated period (2002-2021) there were 247 patients with a primary diagnosis of pulMZL. The diagnosis was established in 29.5% of patients in the first, 21.9% in the second, 21.3% in the third and in 27.3% in the fourth quarter of the year. There were no statistically significant differences but a trend with a peak in the winter months and a trough during summer. Stratifications of the cases by age or sex did not reveal any statistically significant seasonal differences. There were also no significant differences between the periods before and during the COVID-19 pandemic.

### Hairy cell leukemia

Of the 451 patients with HCL 25.1% were diagnosed in the first quarter of the year, 27.8% in the second, 24.0% in the third and 23.1% in the fourth. There was a noticeable incidence peak in the first six months of the year but however no significant statistical differences between the various quarters of the year.

Likewise, stratification of the cases by age or sex revealed no statistically significant seasonal differences. There were also no significant differences between the periods before and during the COVID-19 pandemic.

### Diffuse large B-cell lymphoma

In the investigated period (2015 and 2019) there were 4577 cases with newly diagnosed DLBCL.

The diagnosis was established in 24.4% of patients in the first, 25.8% in the second, 26.0% in the third and in 23.8% in the fourth quarter of the year. Thus no statistically significant differences between the various quarters of the year were noted. Stratifications of the cases by age or sex did not reveal any statistically significant seasonal differences.

**Figure 7:**
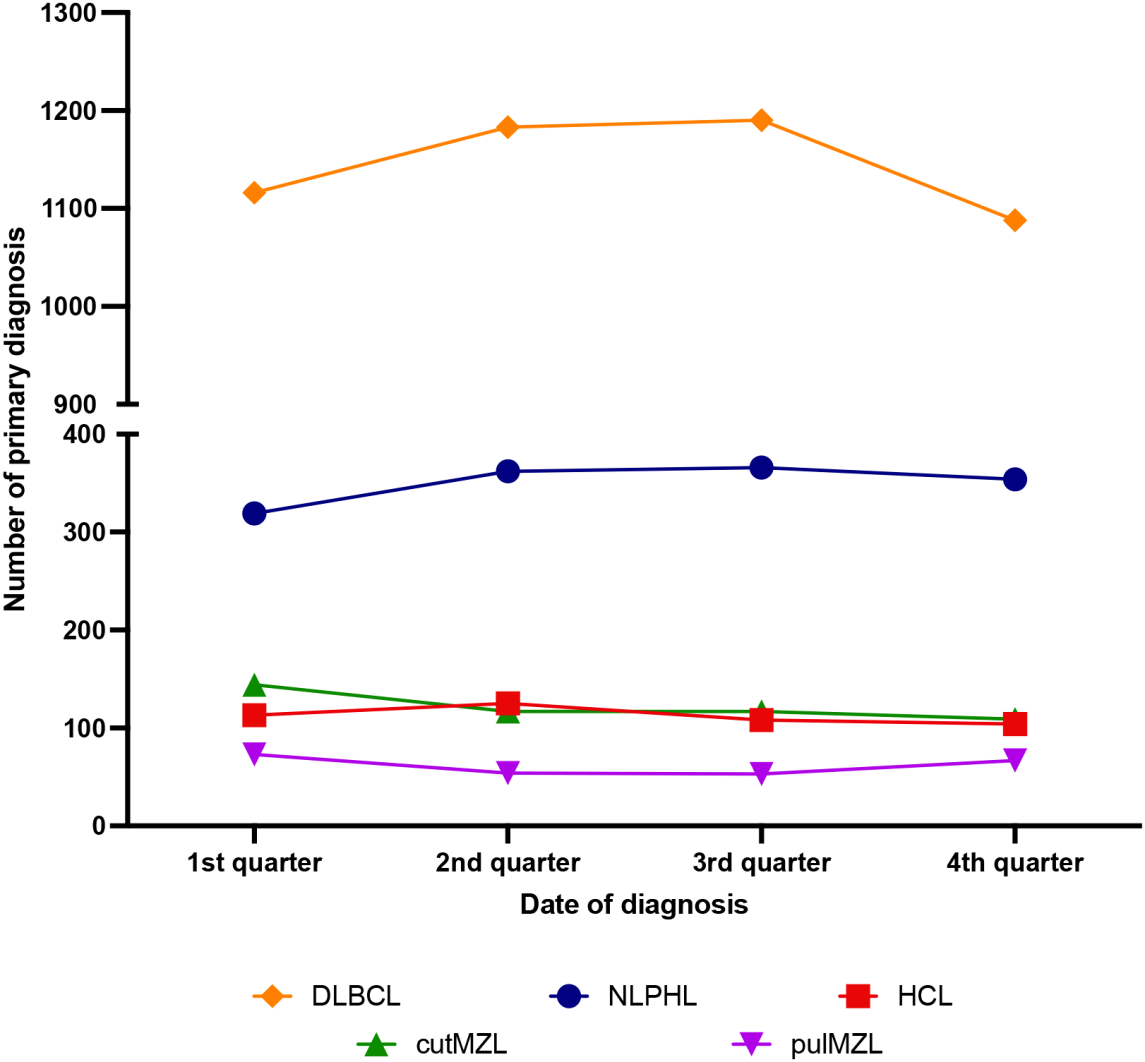
Seasonal distribution of newly diagnosed diffuse large B-cell lymphoma (DLBCL), nodular lymphocyte predominant (NLPHL), pulmonary marginal zone lymphoma (pulm MZL), cutaneous marginal zone lymphoma (cut MZL) and hairy cell leukemia (HCL) cases. The time intervals differed between the various entities (for details see text).

The results of DLBCL, NLPHL, cutaneous and pulmonary MZL and of HCL are summarized on **Figure 7**.

## Discussion

So far, the published studies on seasonal occurrence of lymphomas have mainly dealt with Hodgkin lymphomas, particularly with CHL, and in a much lesser extent with NHL. These studies were based on data from variably sized cancer registries, the largest being the SEER database, which has been used only in two studies (7, 9). The SEER database is a publicly available, federally funded cancer reporting system representing a collaboration between the US Centers for Disease Control and Prevention, the National Cancer Institute, and regional and state cancer registries. SEER data are national, with information from 18 states representing all regions of the USA capturing approximately 28% of the US population. Of course, the SEER database has the enormous strength of large size, a population basis and uniformly collected data. Our study has followed a different approach as we used the files from one of Germany’s six lymphoma reference centers, located at the Institute of Pathology of the University of Würzburg. As the reference centers receive paraffin blocks of suspected lymphoma cases for a second opinion usually within one week after the material has reached the local pathologists located in different parts of Germany, we assumed that we could be able to achieve representative data using the month of receipt of lymphoma specimens for our study. By following this approach, we could reproduce the findings of the largest study regarding a seasonality of CHL. That study used data from the SEER registry and found, that the overall incidence pattern of CHL diagnosed in the northern hemisphere peaks around March and shows a trough around September (7). Our data based on 8.038 cases diagnosed during the period 2007–2021 showed a peak in the first quarter and a trough in the third quarter of the year.

Regarding seasonal incidence patterns in correlation with the different histologic subtypes of CHL, Douglas and coworkers demonstrated a seasonality for the nodular sclerosis subtype with a peak in March (3). Neilly et al. also found a significant seasonal incidence pattern for the nodular sclerosis and mixed cellularity subtypes with a peak in March (1). Borchmann and coworkers reported similar results for the nodular sclerosis, mixed cellularity and additionally for the lymphocyte depleted subtypes (7). We could also reproduce these findings by showing that among our cases the nodular sclerosing subtype displayed the same seasonal pattern as all CHL cases. Our negative results regarding seasonal correlations with the other CHL subtypes may be due to the fact that they were less frequently represented than nodular sclerosis in our cohort.

Few studies on seasonality and CHL have also dealt with correlations of seasonal incidence patterns of CHL with the patient’s age. Chang et al. performed a stratified analysis by age groups and detected a statistically significant excess of HL diagnoses in February among the age group of 14–49 years (5). Borchmann et al. observed that the overall incidence peak around March was more pronounced in the age groups 20–29 and 30-39 (7). In line with that, the observed seasonal CHL incidence differences in our cohort with a peak in the first quarter of the year, were mainly driven by the younger age groups (0–19 and 20–39 years).

As we could reproduce almost all findings regarding the seasonal fluctuation of CHL diagnosis obtained on more extensive databases than ours, we wanted to address, whether the seasonal pattern observed by us and others might correlate with a causative infection. Several studies have raised the hypothesis that seasonality in HL (1, 5, 10) and NHL of B-lineage (9) might be caused by an infectious etiology. CHL lymphoma is particularly attractive to shed some light in this context, as a significant number of cases is associated with an EBV infection of the neoplastic cells. While the SEER database used by Borchmann et al. did not supply the information of an EBV association, the cases of our collective have been studied for this as a part of the routine diagnostic procedure. In particular all CHL had been analyzed for their association with EBV either by detection of EBV-encoded RNAs (EBER) that are present in all latently EBV-infected cells or by immunohistochemical labelling for the latent membrane protein-1 (LMP-1) that characterizes EBV-latency type II found in CHL. Our data convincingly show that EBV-associated CHL do not show a seasonal pattern but a continuous decline in their incidence during the year. Notably, the mixed cellularity CHL-subtype exhibited a similar curve as the EBV-positive CHL cases. This is apparently due to the fact, that our mixed cellularity cases were more frequently EBV-associated than the nodular sclerosing subtype (54.1% EBV-positive mixed cellularity vs. 17.4% EBV-positive nodular sclerosis). Our data also show that the seasonal pattern observed in the younger age groups (0-39) is similar to that observed in the EBV-negative cases. Earlier data also have shown that EBV-negative CHL are more frequent among younger patients (27).

As already stated, only one SEER-based study shows a peak in March and April in a broadly defined “B-cell-NHL” category in patients below the age of 65 years (9). Due to the fact that the most frequent B-cell lymphoma that also made out a significant proportion of the cases in the mentioned study is DLBCL, we surveyed all newly diagnosed DLBCL cases within a 5 year interval for a possible seasonal association. Our data could not reproduce those of Koutros et al. (9), as no seasonality at DLBCL diagnosis could be observed. Also additional correlations with age and sex of the patients did not reveal significant findings. As a next step, we wanted to determine whether B-cell lymphomas with suspected infectious etiology do show any seasonal association. For this reason we investigated primary MLZ of the skin (suggested association with *Borrelia burgdorferi*) *(* 20) and the lung (suggested association with *Achromobacter xylosoxidans*) (16) as well as HCL, which exhibits some indirect evidence for an infectious etiology (mutated *IGH* genes with evidence of antigen selection/T-bet expression) (21, 25). Our patient population comprising 487 cases of cutMZL, 247 cases of pulMZL and 451 cases of HCL, did not show any significant seasonal association although available representative data for Borreliosis show a peaked disease onset in July (28, 29). Thus, our overall data implies that neither a direct infection of neoplastic cells by a virus nor a chronic stimulation by a pathogen or another chronic inflammatory condition can lead to seasonal fluctuation in lymphoma diagnosis. Of course, we cannot exclude that our negative findings might be caused by the low case numbers of the lymphoma entities investigated. Our findings are however in line with the suggestion of Borchmann et al., that an infectious etiology for CHL does not explain the observed seasonal differences in mortality (7) and also contradicts the finding that the seasonal incidence pattern in CHL is stronger in age groups with a fairly high proportion of EBV-negative cases (27).

What may be causes of the observed seasonal pattern in CHL? It might be possible that CHL diagnoses peaked in the first quarter of the year following a clinical work-up for symptoms of a springtime or lingering wintertime infection that led to the incidental discovery of the existing lymphoma. Alternatively, patients with subclinical CHL might be more susceptible to developing infections during winter and spring, leading to a more frequent detection during this period. Repeated infections during the wintertime might also have led to an active stimulation of the B-cell receptor signaling pathway that can be a predisposing condition for lymphomagenesis (30). Another possibility might be that the patients have postponed attending to signs and symptoms of CHL during the holiday months of December and January. Similarly, the decrease in CHL diagnoses among young adults in the third quarter of the year could be due to patients’ delay and/or physicians’ absence during summer vacation. The most attractive hypothesis regarding the cause of the seasonal patterns of CHL is the correlation with the vitamin D levels mediated by ultraviolet radiation as proposed by Borchmann et al. (7). The authors found out that the seasonal incidence pattern observed in CHL occurring at higher latitudes exactly resembles the seasonal pattern of vitamin D levels in humans (31). A low vitamin D level is known to be a risk factor for developing and also suffering from poor prognosis in certain hematologic malignancies (32, 33).

Another aspect that we investigated was whether the COVID-19 pandemic influenced the observed seasonal patterns of CHL. Our analysis of the period 2020–2021 could not identify any significant differences but a trend of an increased incidence in the second half of the year. This might well be due to the fact, that the patients postponed medical consultation in order to avoid COVID-19-infection. It might be also that the preventive measures (practicing hand hygiene, consistently and correctly wearing a mask, improving air circulation and keeping distance when possible) have already reduced the incidence not only of infectious diseases but also of medical consultations leading to reduced detection of existing lymphoma (34). Our data might be however not representative as we have studied only the first 2 years of the pandemic. Additional follow-up analysis when more post-pandemic data are available is certainly needed and will be performed in the future.

In conclusion, we could reproduce the findings on the seasonal occurrence patterns in CHL and found that they do not correlate with an EBV infection of the neoplastic cells. Furthermore, we did also not observe any seasonality in the investigated B-NHL entities with presumed infectious etiology.

## Data Availability

The raw data supporting the conclusions of this article will be made available by the authors, without undue reservation.
